# Estimation of Sound Transmission Loss in Nanofiber Nonwoven Fabrics: Comparison of Conventional Models and the Simplified Limp Frame Model

**DOI:** 10.3390/nano13222947

**Published:** 2023-11-14

**Authors:** Shuichi Sakamoto, Tsukasa Hasegawa, Koki Ikeda

**Affiliations:** 1 Department of Engineering, Niigata University, Ikarashi 2-no-cho 8050, Nishi-ku, Niigata City 950-2181, Japan; 2 Graduate School of Science and Technology, Niigata University, Ikarashi 2-no-cho 8050, Nishi-ku, Niigata City 950-2181, Japan

**Keywords:** nanofiber nonwoven fabrics, transmission loss, limp frame model, parameter study

## Abstract

Although the sound absorption coefficients of conventional and nanofiber nonwoven fabrics (NF-NWFs) have been the subject of many previous studies, few studies have considered the estimation of transmission loss. Reported herein is an experimental and theoretical study into estimating the transmission loss of NF-NWFs using four estimation models, i.e., the Rayleigh, Miki, and Komatsu models, and the simplified limp frame model (SLFM), with the model results compared against the experimental data. The transmission loss of the NF-NWF was determined from the propagation constant, and characteristic impedance was calculated using the estimation model and the transfer matrix method. The validity of each estimation method was examined by comparing its estimated values with the experimental values measured using a four-microphone impedance measurement tube. The proposed SLFM is more suitable for estimating the transmission loss of NF-NWFs than the conventional Rayleigh, Miki, and Komatsu models.

## 1. Introduction

Poroelastic materials [1], such as fiber materials, are used in various fields, such as the automotive industry [2,3], because of their high sound-absorbing performance at medium to high frequencies. In particular, nanofiber nonwoven fabrics (NF-NWFs) comprising fibers with diameters of 1 µm or less are attracting attention as lightweight materials with a high sound-absorbing performance [4,5,6].

While previous studies extensively covered the sound absorption coefficient of conventional and nanofiber NWFs [7,8,9], measurements involving parameters such as PVP gel porosity, ventilation resistance, and sound absorption have also been conducted [10]. However, only a few studies have estimated the transmission loss [11,12]. For example, speech is attenuated by nonwoven masks used to protect against infectious diseases, resulting in reduced intelligibility [11,12]. In the medical field, the double penetration of the voice, due to the nonwoven mask worn by the speaker and protective clothing worn by the listener, reduces the intelligibility of the conversation. Furthermore, nonwoven filters are also used in vacuum cleaners, air purifiers, air conditioners, and air cleaners for internal combustion engines, and sound absorption and insulation are known secondary effects of filters. Recently, the use of NF-NWFs with fiber diameters of 1 µm or less has been expanding, such as those used in nanofiber masks [13]. In mask applications, the filter should exhibit high efficacy, meet ventilation resistance standards, and maintain low acoustic transmission loss. Conversely, filters used for machinery ideally require high filtration efficiency, low ventilation resistance, and high transmission loss. These applications present conflicting performance requirements, particularly in terms of transmission loss. Hence, assessing transmission loss is crucial for determining the most suitable application.

The Rayleigh [14], Miki [15], and Komatsu models [16] are used for estimating the acoustic properties of fiber materials. These models are formulated with the flow resistance of the fiber material as a variable that is reflected in the characteristic impedance and propagation constant of the material. However, it is necessary to examine the applicability of these models to estimate the transmission loss of NF-NWFs in practice.

In the above context, the limp frame model (LFM) [17,18] has attracted attention as a model for estimating the acoustic properties of NF-NWFs. In particular, previous research has shown that the simplified LFM (SLFM) is useful for estimating the sound absorption coefficient of NF-NWFs [19]. This model uses only two Biot parameters [20,21], i.e., flow resistance and bulk density, which are relatively easy to measure and, in this way, the characteristic impedance and propagation constant are obtained more simply than with the LFM. 

This study aims to predict transmission loss using the SLFM for NF-NWFs produced through various manufacturing processes that use different materials and structures. In addition, the samples include microfibrillated cellulose nonwoven fabrics (MFC-NWFs) made from cellulose fibers. Cellulose is widely used in structural materials with diverse applications, including acoustic ones [22,23,24]. However, only a few studies have investigated the transmission loss of materials in which cellulose is acoustically incorporated [25]. Therefore, this experiment seeks to predict the transmission loss of MFC-NWFs.

This paper demonstrates the utility of the SLFM for estimating the transmission loss of NF-NWFs by conducting a parameter study on six Biot parameters, namely, thermal characteristic length, viscous characteristic length, tortuosity, porosity, flow resistivity, and bulk density, in the LFM. Reported herein is also an experimental and theoretical study into estimating the transmission loss of NF-NWFs using four estimation models, i.e., the Rayleigh, Miki, and Komatsu models, and the SLFM, with the model results compared against the experimental data.

## 2. Samples and Measurement Setup

### 2.1. Samples

**Figure 1:**
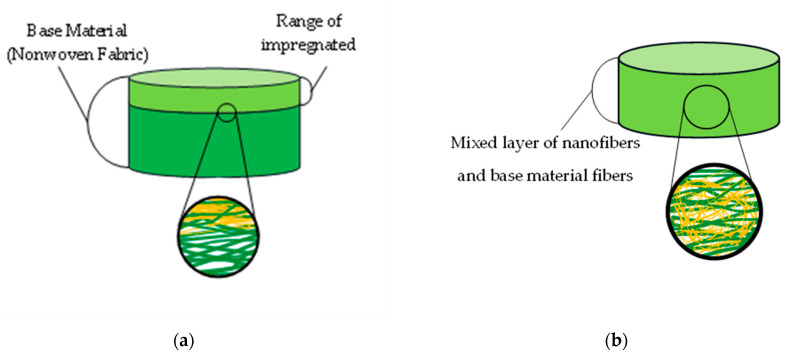
Schematics of samples. (**a**) Samples prepared via electrospinning and Sample 2. (**b**) Tenma 1–4. Although Sample 2 is produced using a different method than the electrospinning technique, it exhibits structural similarities.

**Figure 2:**
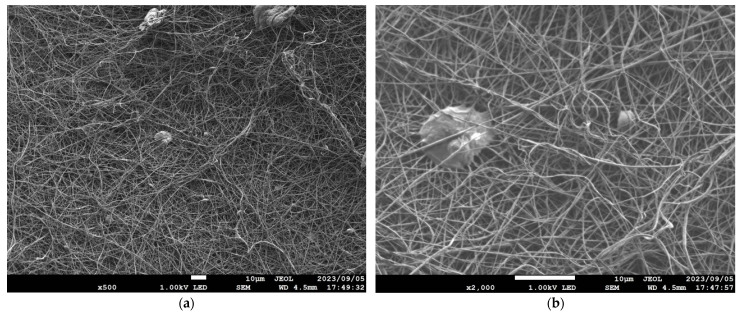
Microscopy observations (JSM-7800F Prime FE-SEM, JEOL). Shinwa 1-1 at (**a**) 500× and (**b**) 2000× magnification.

**Figure 3:**
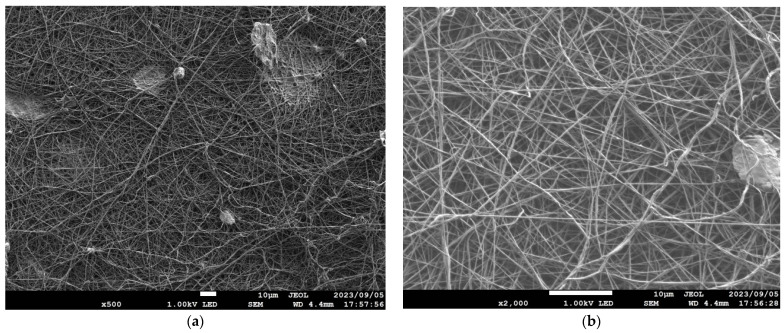
Microscopy observations (JSM-7800F Prime FE-SEM, JEOL). Shinwa 1-2 at (**a**) 500× and (**b**) 2000× magnification.

**Figure 4:**
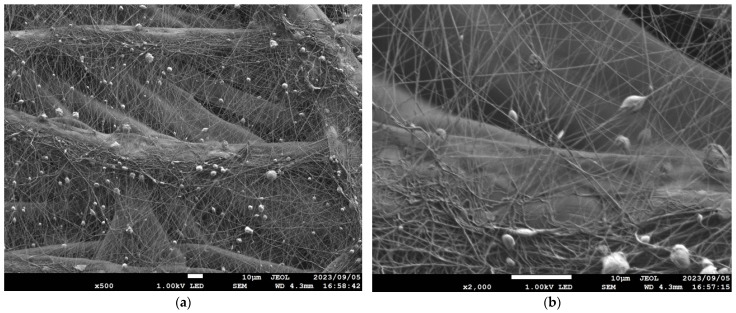
Microscopy observations (JSM-7800F Prime FE-SEM, JEOL). Sample A at (**a**) 500× and (**b**) 2000× magnification. Notably, the substrate fibers and nanofibers are clearly visible.

**Figure 5:**
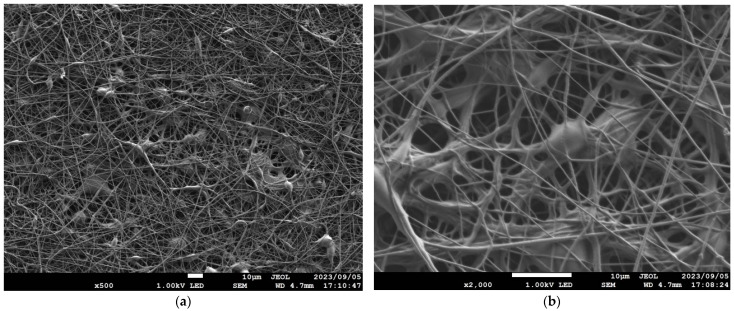
Microscopy observations (JSM-7800F Prime FE-SEM, JEOL). Sample 1 at (**a**) 500× and (**b**) 2000× magnification.

**Figure 6:**
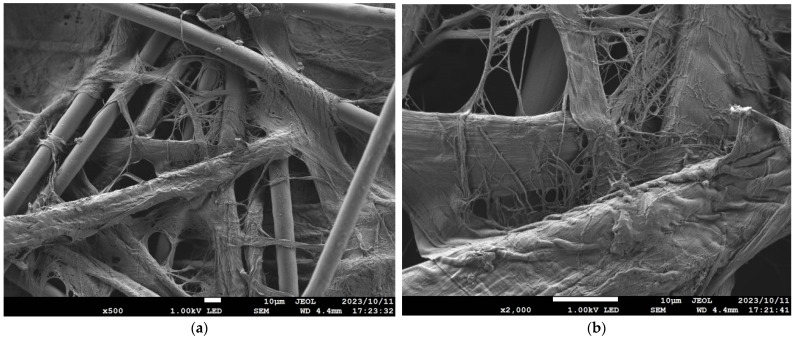
Microscopy observations (JSM-7800F Prime FE-SEM, JEOL). Tenma 4 at (**a**) 500× and (**b**) 2000× magnification. The fibers that appear to adhere to the base material fibers are MFC.

Table 1 details the NF-NWFs used in the experiments and in estimating transmission loss, Table 1 details the base materials (i.e., the NWFs used as the base on which the nanofibers were placed), and Figure 1a,b show the structures of the NF-NWFs schematically. Shinwa 1-1 (Shinwa Co., Ltd., Takatsuki, Osaka, Japan), Shinwa 2-1, Sample A, Sample 1 and Tenma 4 (Tentok paper Co., Ltd., Fuji, Shizuoka, Japan) were observed using a scanning electron microscope (JSM-7800F Prime FE-SEM, JEOL, Tokyo, Japan), and their micrographs, taken at magnifications of 500× and 2000×, are shown in Figure 2, Figure 3, Figure 4, Figure 5 and Figure 6, respectively. To ensure a broad scope, samples were sourced from several major Japanese manufacturers of NF-NWFs.

NF-NWF Samples A–F, Shinwa 1-1–1-4, Shinwa 2-1–2-4, and Sample 1 were fabricated using electrospinning [26] (Structure; Figure 1a).

Samples A–F were made of polyethylene terephthalate (PET) for the base NWF and polyvinylidene fluoride resin (PVDF) for the nanofibers. Samples A and B and Samples C–F were manufactured using different base materials; Samples A and B had Sample G as their base material, and Samples C–F had Sample H as their base material (see Table 1 for specifications).

For Shinwa 1-1–1-4, the base NWF was made of polypropylene (PP), and the nanofibers were made of PVDF. Shinwa 1-5 is used for these base materials. For Shinwa 2-1–2-4, the nanofibers were made of PVDF, but the base material was a core–sheath fiber, where the core part was made of PP, the sheath part was made of polyethylene (PE), and the ratio of PP to PE was 1:1; the base material was Shinwa 2-5. In Sample 1, the base material was PET, and the nanofibers were made of polyethersulfone (PESU).

The following NF-NWFs were not produced via electrospinning. In Sample 2, a needlepunched NWF consisting of fibers, including sea-island structure fibers, was prepared, and this NWF was subjected to an alkaline extraction process to produce NF-NWF (Structure; Figure 1a). Tenma 1–4 were MFC-NWFs (Structure; Figure 1b); the nanofibers were composed of cellulose, the base material was PP, and the ratio of cellulose to PP was 7:3. Here, nano-sized fibers can be seen from the micrographs. For instance, according to JIS T 9001, medical masks should have a ventilation resistance of up to 0.111 kPa·s/m. In this study, Shinwa 1-4 and Shinwa 2-4 met this standard.

### 2.2. Equipment for Measuring Transmission Loss

**Figure 7:**
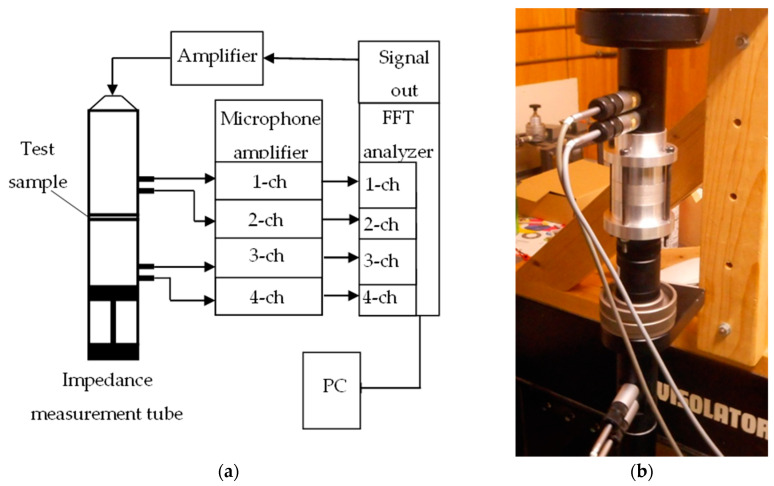
Four-microphone impedance tube for measuring transmission loss: (**a**) Schematic; (**b**) Photograph.

Figure 7a,b show a schematic and photograph depicting the experimental setup for measuring transmission loss. A sample was placed in a Brüel & Kjær (Teknikerbyen, Virum, Denmark) Type 4206 four-microphone impedance measurement tube, and a sinusoidal sound wave was produced from a speaker attached to a signal generator incorporated into a fast Fourier transform (FFT) analyzer. The cross-spectrum between the sound pressure signals of the four microphones attached to the impedance tube was measured using the FFT analyzer, and transmission loss was calculated using the measured cross-spectrum in accordance with ASTM E2611-09. The NWF being measured was supported without tension at a specified position in the impedance tube.

The critical frequency for the formation of plane waves differs depending on the inner diameter of the acoustic tube. In this study, a small tube with an inner diameter of 29 mm was used, so the measurement range was 500 to 6400 Hz. The voltage of the signal input to the speaker was 2.0 V_p-p_.

### 2.3. Equipment for Measuring Ventilation Resistance

**Figure 8:**
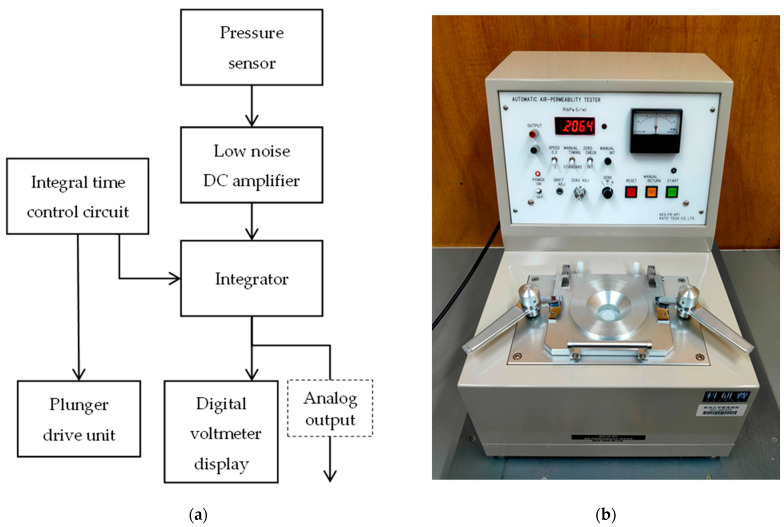
Air permeability tester (KES-F8-AP1, Kato tech Co., Ltd.): (**a**) Schematic; (**b**) Photograph. A plunger-type air permeability tester was used in this study, enabling measurements even in scenarios with high ventilation resistance.

An air permeability tester (KES-F8-AP1, Kato Tech Co., Ltd., Kyoto, Japan) was used in the experiment (Figure 8a,b). The following steps were performed for measuring the ventilation resistance. A sample was placed over the ventilation hole, through which air was released at a fixed flow rate into the atmosphere and then sucked back through the sample by the piston movement of the plunger. The pressure drop across the sample was measured using a semiconductor differential pressure gauge and averaged over 3 s for each half cycle of release and suction. Ventilation resistance is expressed as the pressure divided by the amount of airflow, and so, because the pressure is proportional to the ventilation resistance, the latter was calculated using the pressure drop. In this study, the piston speed was set to 2.0 cm/s, and the output sensitivity and full scale were set to 20 Pa/V and 200 Pa/10 V, respectively.

## 3. Theoretical Analysis

### 3.1. Conventional Estimation Model for Nonwoven Fabrics

The theoretical models that are examined and compared in this study are described below.

The Rayleigh model [14] is a model that uses flow resistivity, and its porosity and tortuosity are not considered. It is a classical estimation model that approximates a porous material as an aggregate of capillaries, and it is still used as an estimation model for porous materials in the numerical finite-difference time-domain analysis method.

The Miki model [15] is used for estimating the acoustic behavior of porous materials, and it has been shown to be effective even outside the effective frequency range of the Delany–Bazley model [27], especially for multiple layers.

The Komatsu model [16] estimates the acoustic properties of brittle materials solely from their flow resistivity, and it has been shown to be more effective than conventional models, especially for low-density fiber materials. 

The LFM [17,18] is an estimation model that can be applied to poroelastic materials with soft skeletons, and it has been shown to be effective in estimating the sound absorption coefficient of NF-NWFs [19]. Assuming that the stiffness of the poroelastic material skeleton is negligible and that the elasticity is very low, the sound propagating through the skeleton propagates simultaneously with the air-borne sound in the voids, and they can be treated as interacting with each other. The LFM uses six Biot parameters [20,21] to estimate the acoustic properties. The LFM and SLFM are described in Section 3.2.

### 3.2. Limp Frame Model and Its Simplification

The LFM [17,18] uses the six Biot parameters given in Table 2 to estimate acoustic characteristics. Table 2 also shows the values of the Biot parameters for Sample A.

**Figure 9:**
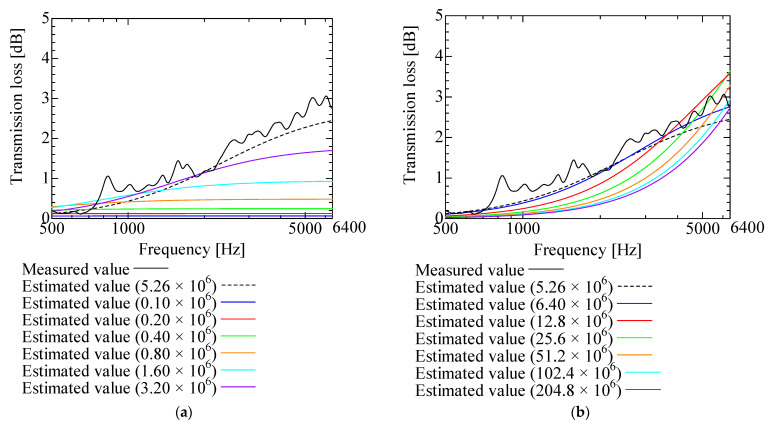
Variation of estimated transmission loss with flow resistivity: (**a**) Flow resistivity 0.10 × 10^6^–3.20 × 10^6^ and 5.26 × 10^6^; (**b**) Flow resistivity 6.40 × 10^6^–204.8 × 10^6^ and 5.26 × 10^6^.

**Figure 10:**
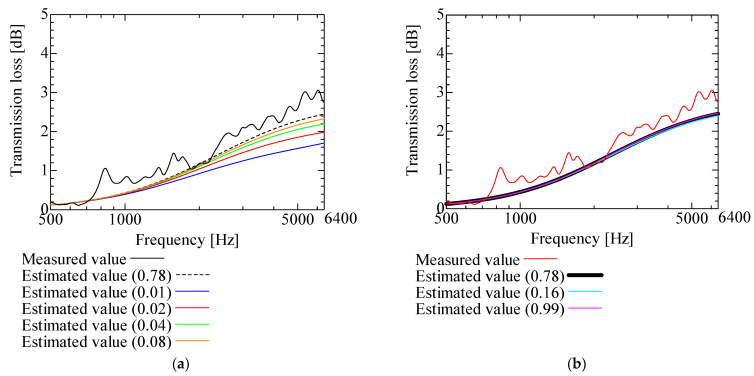
Variation of estimated transmission loss with porosity: (**a**) Porosity 0.01, 0.02, 0.04, 0.08, and 0.78; (**b**) Porosity 0.16, 0.99, and 0.78.

**Figure 11:**
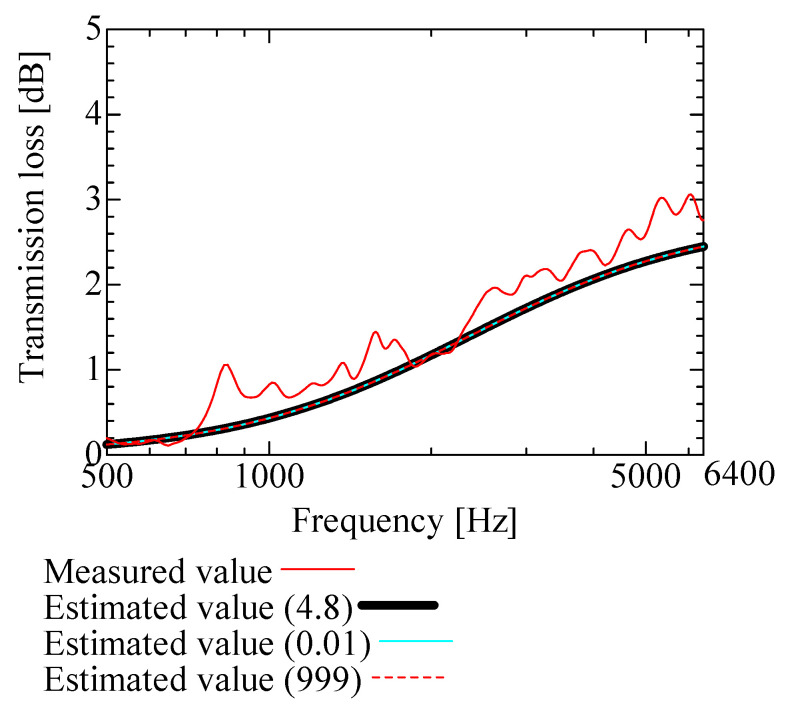
Variation of estimated transmission loss with viscous characteristic length: viscous characteristic lengths of 0.01, 999, and 4.8.

**Figure 12:**
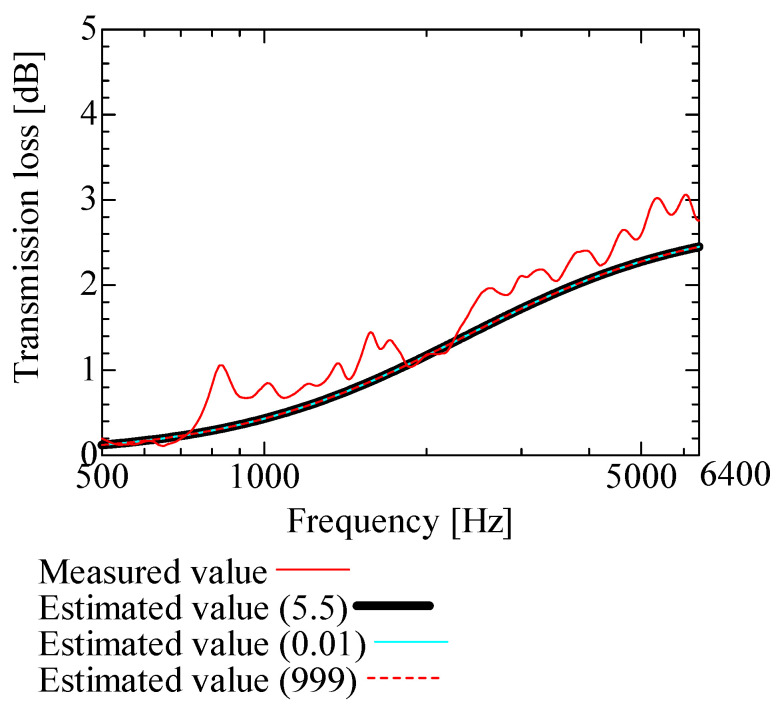
Variation of estimated transmission loss with thermal characteristic length: thermal characteristic lengths of 0.01, 999, and 5.5.

**Figure 13:**
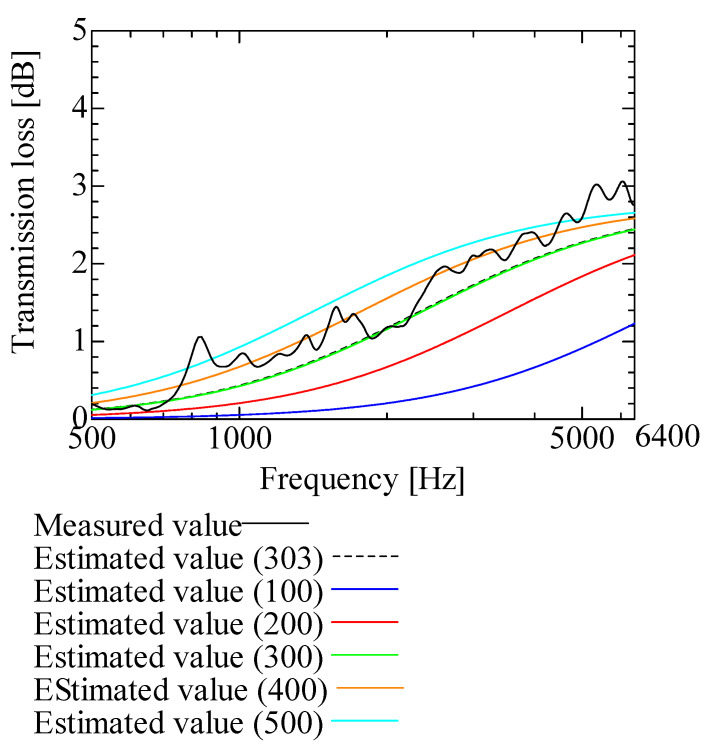
Variation of estimated transmission loss with bulk density: bulk densities of 100, 200, 300, 400, 500, and 303.

Figure 9, Figure 10, Figure 11, Figure 12 and Figure 13 show how the transmission loss of the NF-NWFs changes when each of the following parameters is varied. The values shown in Table 2 were used for the parameters other than those to be varied. Since the tortuosity of fiber-based materials is approximately 1.0, the study of the tortuosity parameter was omitted [28]. Figure 9 shows that, with increasing flow resistivity, the transmission loss tends to increase and then decrease. Figure 10 shows that, with increasing porosity, the transmission loss tends to initially increase, but when the porosity exceeds 0.16, the transmission loss hardly changes. Figure 11 and Figure 12 show that the transmission loss is independent of the viscous and thermal characteristic lengths. Finally, Figure 13 shows that, with increasing bulk density, the transmission loss tends to increase.

The above results indicate that the main Biot parameters, when estimating the transmission loss of NF-NWFs using the LFM, are flow resistivity, porosity, and bulk density.

### 3.3. Simplified Limp Frame Model

The results in Section 3.2 show that transmission loss can be estimated using the SLFM [16,17,18], which involves only the flow resistivity, porosity, and bulk density. This is similar to previous work involving the sound absorption coefficient [18]. In the SLFM, the effective density [Equation (1)] and effective volume modulus [Equation (4)] are given by

(1) $$\tilde{\rho }\left(\omega \right)=\frac{{\tilde{\rho }}^{\prime }\left(\omega \right)\rho_{t}-\rho_{0}{}^{2}}{\rho_{t}+{\tilde{\rho }}^{\prime }\left(\omega \right)-2\rho_{0}}$$

(2) $${\tilde{\rho }}^{'}\left(\omega \right)=\frac{\rho_{0}}{\phi }\left[1-\frac{\phi \sigma }{j\omega \rho_{0}}\sqrt{1+4j\mu \omega \frac{\rho_{0}}{\sigma^{2}\phi^{2}}}\right]$$

(3) $$\rho_{t}=\rho +\phi \rho_{0}$$

(4) $$\tilde{K}\left(\omega \right)=\frac{\Gamma P_{0}}{\Gamma -\left(\Gamma -1\right){\left[1+\frac{8\kappa }{j\omega }\sqrt{1+\frac{j\omega }{16\kappa }}\right]}^{-1}}$$

and, using the bulk density *ρ* and the true density *ρ_t_*, the porosity *ϕ* is expressed as

(5) $$\phi =1-\frac{\rho }{\rho_{t}}$$

For NF-NWFs, such as those used in this study, if the area density and thickness are *m* and *t*, respectively, then the bulk density *ρ* is expressed as

(6) $$\rho =\frac{m}{t}$$

For the true density *ρ_t_* of the NF-NWF, let *ρ_n_* and *ρ_b_* be the fiber densities of the nanofibers and base material, respectively, and *m*_1_ and *m*_2_ be the area densities of the nanofibers and base material, respectively. Using these, the true density *ρ_t_* is

(7) $$\rho_{t}=\frac{\rho_{n}\rho_{b}m}{\rho_{n}m_{1}+\rho_{b}m_{2}}$$

For the flow resistivity *σ*, let *t* be the sample thickness, *v* be the speed of air in the sample, and ∆*p* be the pressure difference across the sample. Using these, the flow resistivity *σ* is

(8) $$\sigma =\frac{\Delta p}{vt}$$

### 3.4. Propagation Constant and Characteristic Impedance

Next, we describe the propagation constant and characteristic impedance in the SLFM. The propagation constant *γ* is expressed as

(9) $$\gamma =j\omega \sqrt{\frac{\tilde{\rho }\left(\omega \right)}{\tilde{K}\left(\omega \right)}}$$

and the characteristic impedance *Z_c_* is expressed as

(10) $$Z_{c}=\sqrt{\tilde{\rho }\left(\omega \right)\tilde{K}\left(\omega \right)}$$

Then, by substituting the effective density and effective volume modulus derived from Equations (1) and (4) into the right-hand sides of Equations (9) and (10), respectively, the propagation constant and characteristic impedance in the SLFM are obtained.

### 3.5. Calculation of Transmission Loss

Here, we describe how to calculate transmission loss [29] from the transfer matrix. In general, the transfer matrix *T* with respect to sound pressure and volume velocity is expressed as

(11) $$T=\left[\begin{array}{cc} \cosh\left(\gamma l\right) & \frac{Z_{c}}{S}\sinh\left(\gamma l\right)\\ \frac{S}{Z_{c}}\sinh\left(\gamma l\right) & \cosh\left(\gamma l\right) \end{array}\right]=\left[\begin{array}{cc} A & B\\ C & D \end{array}\right]$$

Then, using the four terminal constants *A*, *B*, *C*, and *D*, the transmission loss *TL* of the measured sample is expressed as

(12) $$TL=10\log_{10}\frac{{\left|A+\frac{S_{I_{tube}}}{\rho_{0}c_{0}}B+\frac{\rho_{0}c_{0}}{S_{I_{tube}}}C+D\right|}^{2}}{4}$$

where *S_Itube_* is the cross-sectional area of the impedance measurement tube. Finally, substituting Equations (9) and (10) into Equation (11), the four terminal constants *A*, *B*, *C*, and *D* are obtained, and substituting *A*, *B*, *C*, and *D* into Equation (12) yields the transmission loss of the NF-NWF. 

## 4. Comparison of Measured and Estimated Values

In this section, the transmission loss of NF-NWFs is estimated using the conventional Rayleigh, Miki, and Komatsu models and the proposed SLFM. Figure 14, Figure 15, Figure 16, Figure 17, Figure 18, Figure 19, Figure 20, Figure 21, Figure 22, Figure 23, Figure 24, Figure 25, Figure 26, Figure 27, Figure 28, Figure 29, Figure 30, Figure 31, Figure 32 and Figure 33 show the experimental and estimated results for the NF-NWF samples in order from the sample with the largest transmission loss, and Table 1 shows the corresponding figure number for each sample.

First, the estimates using the Rayleigh model are 1–10 dB larger than the experimental values for all samples, except for Shinwa 1-4. The Rayleigh model estimates show a parallel linear transition and deviate significantly from the experimental trend, and only for Shinwa 1-4 is there good agreement between the experimental and estimated values.

Next, the estimation values calculated using the Miki model are compared with the experimental values. As with the Rayleigh model, these are 1–10 dB larger than the experimental values for all samples except for Shinwa 1-4, and only for that sample is there good agreement between the experimental and estimated values.

Next, the estimates calculated using the Komatsu model are closer to the experimental trend than those of the Rayleigh and Miki models, although there is an error of 2–5 dB compared to the experimental values. However, focusing on the graph slope, there are many samples that differ from the experimental values. Here, the line of the graph is broken for frequency bands outside the applicable range.

Finally, the estimated values calculated using the SLFM are compared with the experimental values. In Figure 14, Figure 15, Figure 16, Figure 17, Figure 18, Figure 19, Figure 20, Figure 21, Figure 22, Figure 23, Figure 24, Figure 25, Figure 26, Figure 27 and Figure 28, these estimated values are closer to the experimental values than those of the other three models, although there are differences of 1–2 dB depending on the sample. The trend of the slope is also similar to the experimental values.

**Figure 29:**
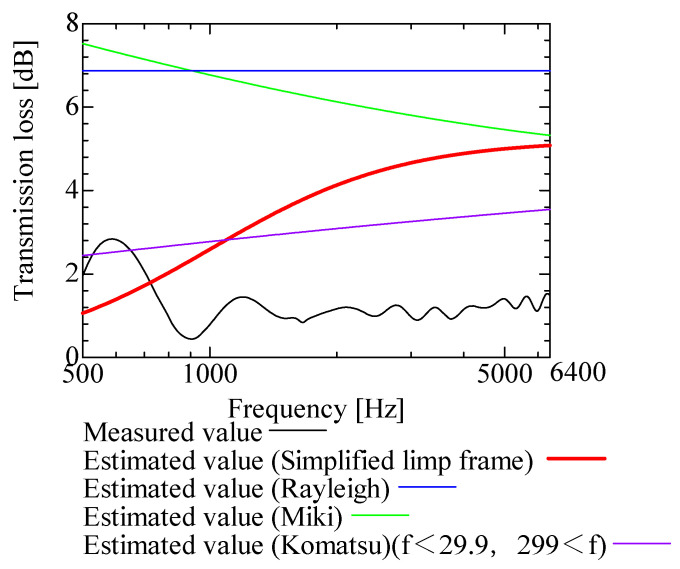
Comparison between experiment and estimates. Sample D, m = 80.12 g/m^2^, t = 230 µm. When the experimental values are <1 dB, none of the estimation models align with the experimental results.

The sample after Figure 29, where the transmission loss is generally less than 1 dB, is supplemented here. As can be seen, when the transmission loss of the NF-NWFs is below ca. 1 dB, it is difficult to estimate using any of the models. Increasing the nanofiber ratio may enhance the transmission loss of NF-NWF. It is commonly perceived that transmission loss and ventilation resistance are typically correlated. However, as observed in Sample 1 and Shinwa 2-1, where the ventilation resistance is similar, the transmission loss in Sample 1 is higher. This suggests that, depending on the material and structural differences, a specific combination of transmission loss and ventilation resistance can be selected for diverse applications.

In general, the SLFM is suitable for estimating the transmission loss of NF-NWFs. However, for Shinwa 1-4, the other models are in better agreement with the experimental data, which is presumably because Shinwa 1-4 resembles the characteristics of ordinary NWFs with low transmission loss.

## 5. Conclusions

The transmission loss of a single NF-NWF was estimated using the SLFM and the conventional Rayleigh, Miki, and Komatsu models. The transmission loss of the NF-NWF was determined using the propagation constant, and characteristic impedance was calculated using the estimation model and the transfer matrix method. The validity of each estimation method was examined by comparing its estimated values with the experimental values measured using a four-microphone impedance measurement tube, and the following conclusions were obtained:

(1) The proposed SLFM is more suitable for estimating the transmission loss of NF-NWFs than the conventional Rayleigh, Miki, and Komatsu models. This study highlights the usefulness of the SLFM in estimating the acoustic insulation performance of NF-NWFs. The SLFM serves as an efficient method for predicting transmission loss to determine the more suitable application between masks and filters, which possess conflicting requirements for transmission loss;
(2) The SLFM can estimate the transmission loss of both MFC-NWFs and other NF-NWFs;
(3) Experiments have revealed the acoustic insulation capabilities of MFC-NWFs constructed from cellulose. This research could be valuable for potentially substituting synthetic fiber filters and masks with those made from cellulose in the future.

It is expected that this study will stimulate the future exploration and utilization of cellulose in acoustic applications and research.

## Figures and Tables

**Figure 14:**
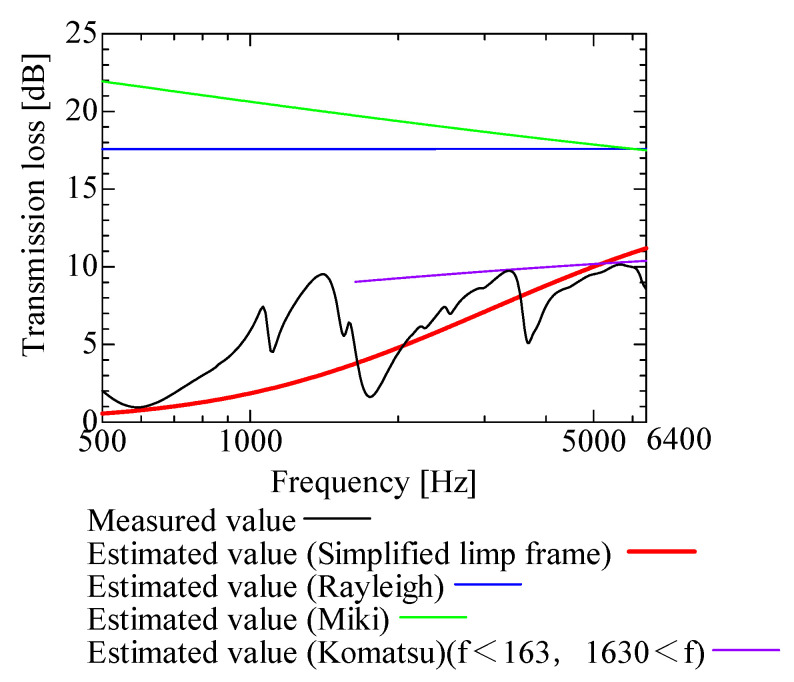
Comparison between experiment and estimates. Sample F, m = 81.1 g/m^2^, t = 230 µm.

**Figure 15:**
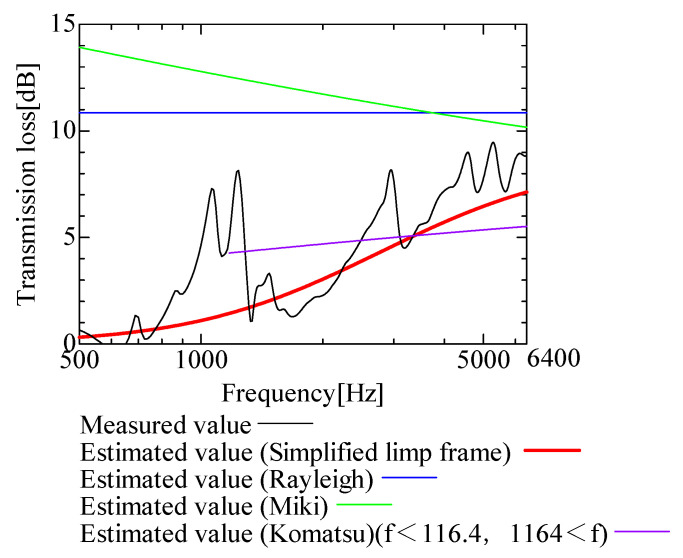
Comparison between experiment and estimates. Tenma 2, m = 49.3 g/m^2^, t = 122.5 µm.

**Figure 16:**
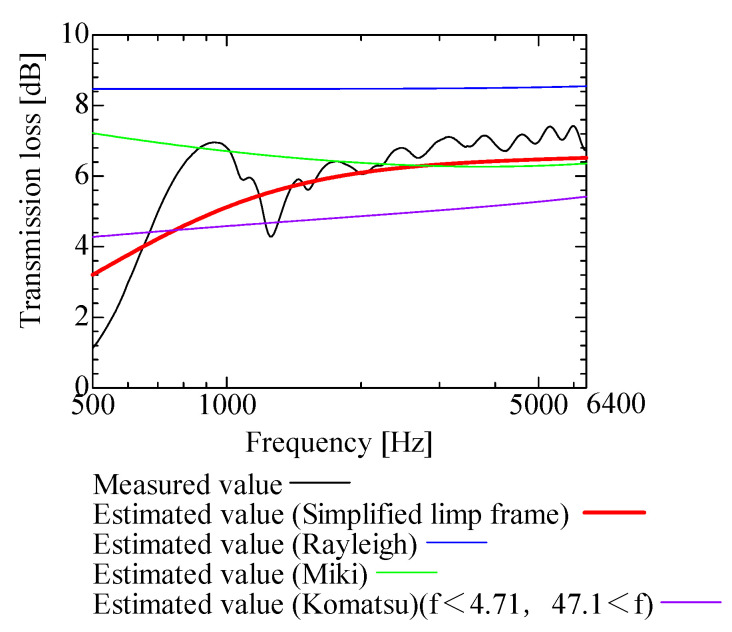
Comparison between experiment and estimates. Sample 2, m = 100 g/m^2^, t = 2000 µm.

**Figure 17:**
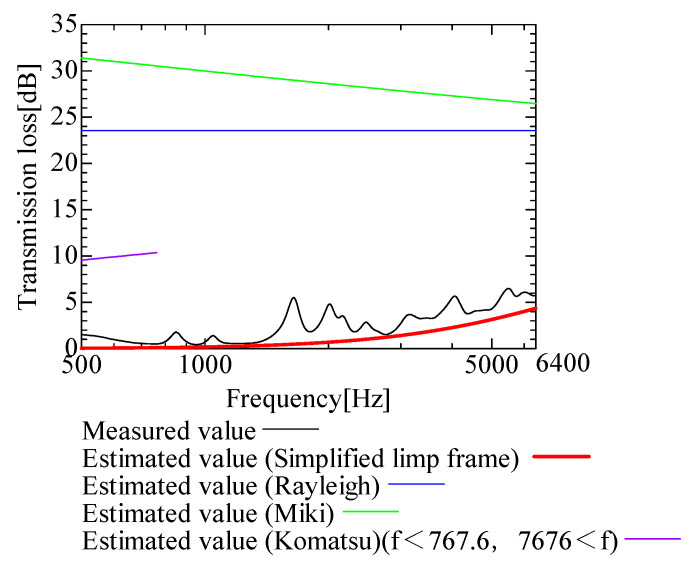
Comparison between experiment and estimates. Tenma 3, m = 25 g/m^2^, t = 104.3 µm.

**Figure 18:**
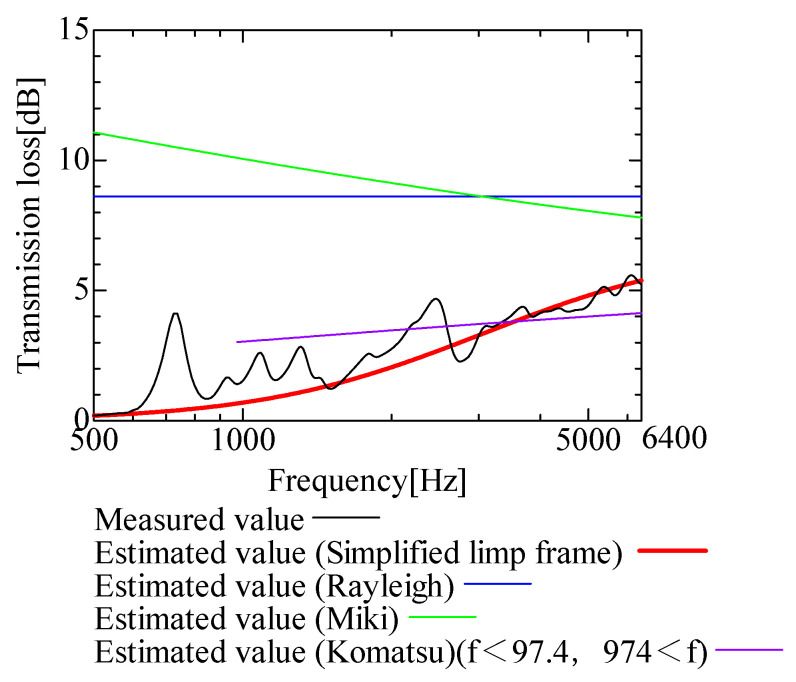
Comparison between experiment and estimates. Tenma 1, m = 34.1 g/m^2^, t = 99.4 µm.

**Figure 19:**
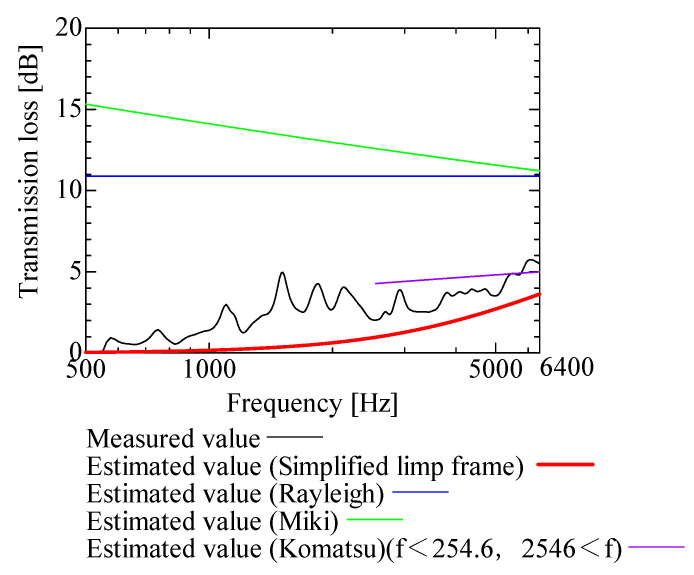
Comparison between experiment and estimates. Sample 1, m = 18 g/m^2^, t = 56 µm.

**Figure 20:**
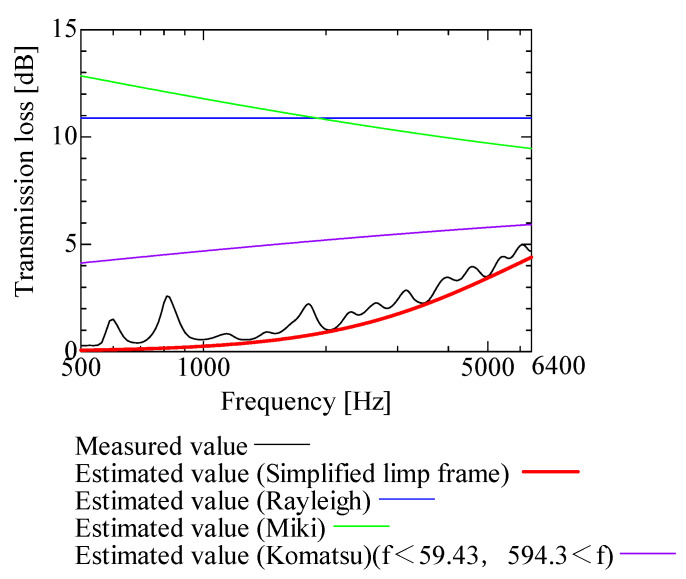
Comparison between experiment and estimates. Shinwa 1-1, m = 22 g/m^2^, t = 240 µm.

**Figure 21:**
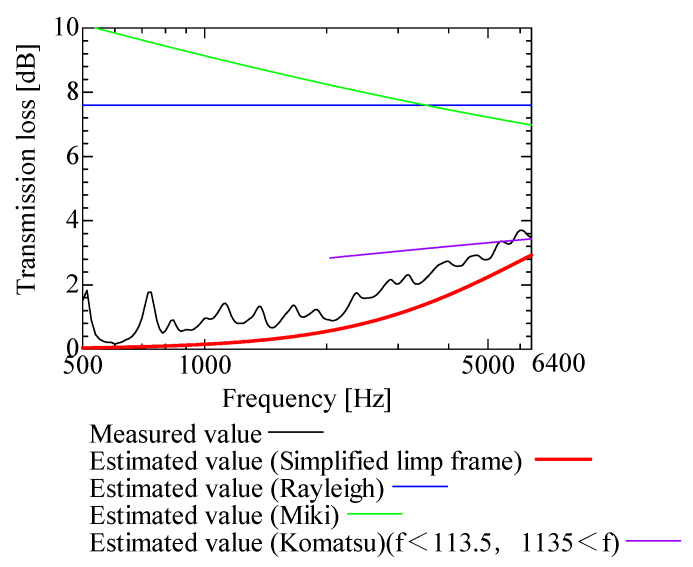
Comparison between experiment and estimates. Shinwa 2-2, m = 14.2 g/m^2^, t = 70 µm.

**Figure 22:**
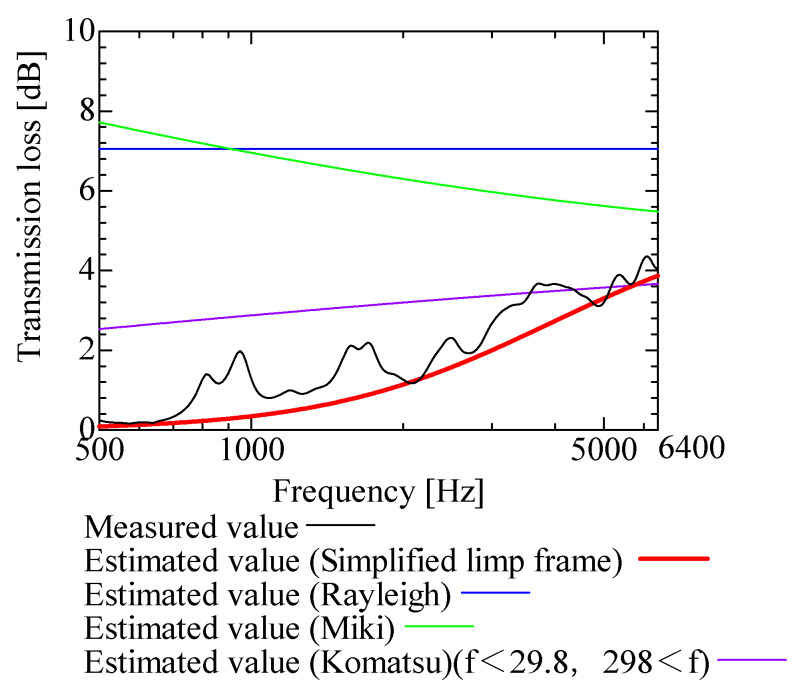
Comparison between experiment and estimates. Shinwa 1-2, m = 21.2 g/m^2^, t = 240 µm.

**Figure 23:**
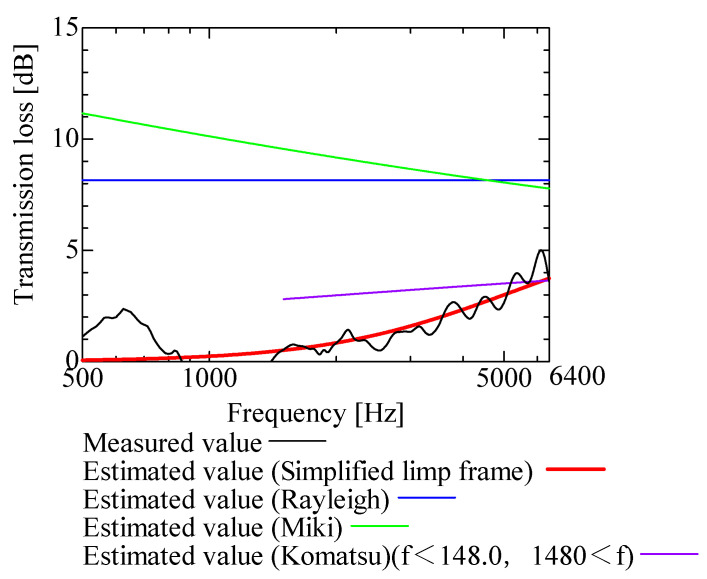
Comparison between experiment and estimates. Sample B, m = 18.6 g/m^2^, t = 60 µm.

**Figure 24:**
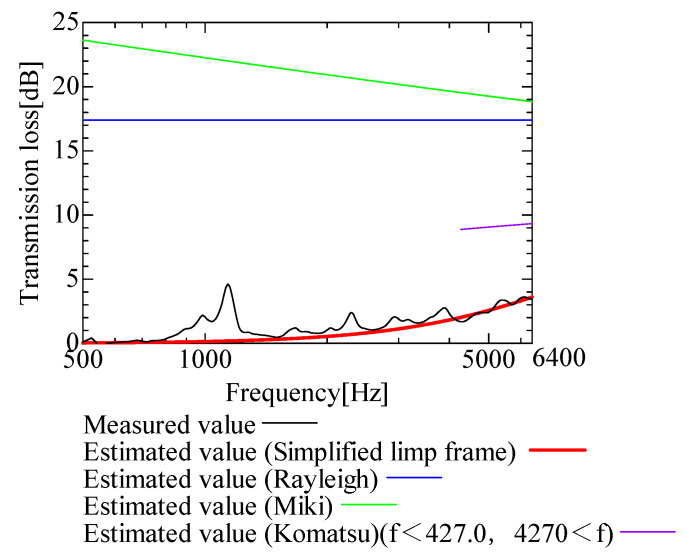
Comparison between experiment and estimates. Tenma 4, m = 20 g/m^2^, t = 85.7 µm.

**Figure 25:**
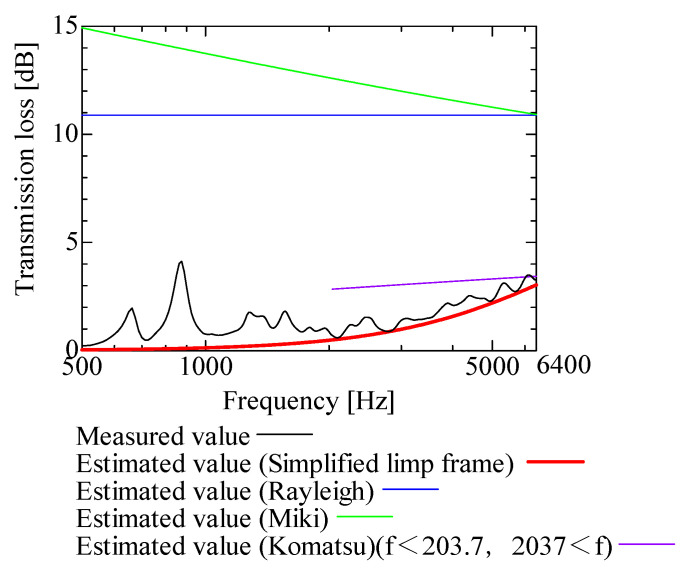
Comparison between experiment and estimates. Shinwa 2-1, m = 15.4 g/m^2^, t = 70 µm.

**Figure 26:**
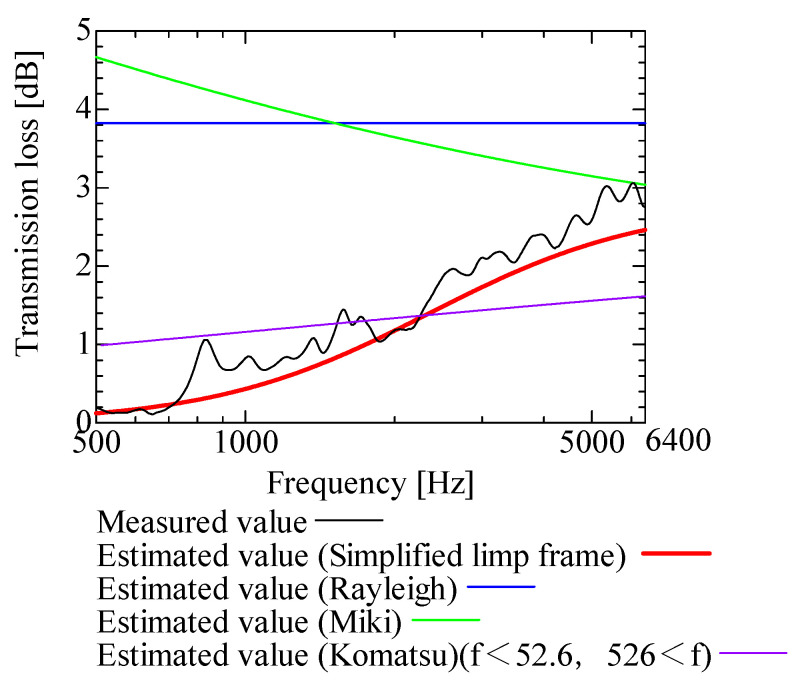
Comparison between experiment and estimates. Sample A, m = 18.2 g/m^2^, t = 60 µm.

**Figure 27:**
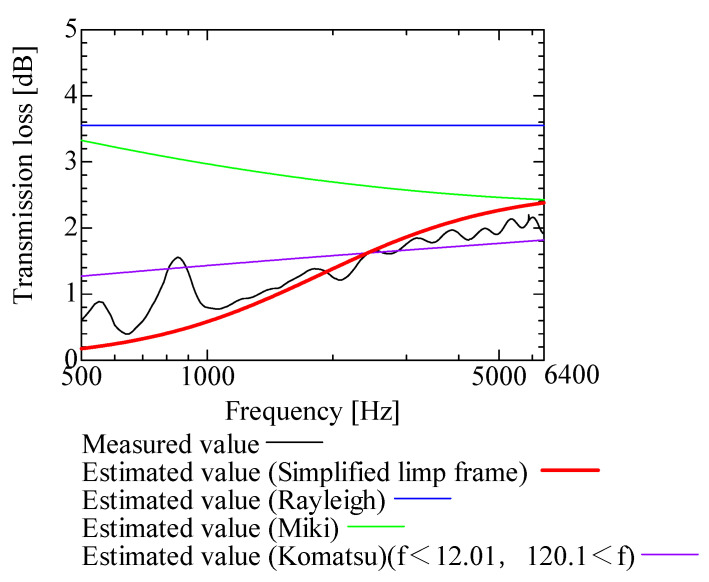
Comparison between experiment and estimates. Shinwa 1-3, m = 20.6 g/m^2^, t = 240 µm.

**Figure 28:**
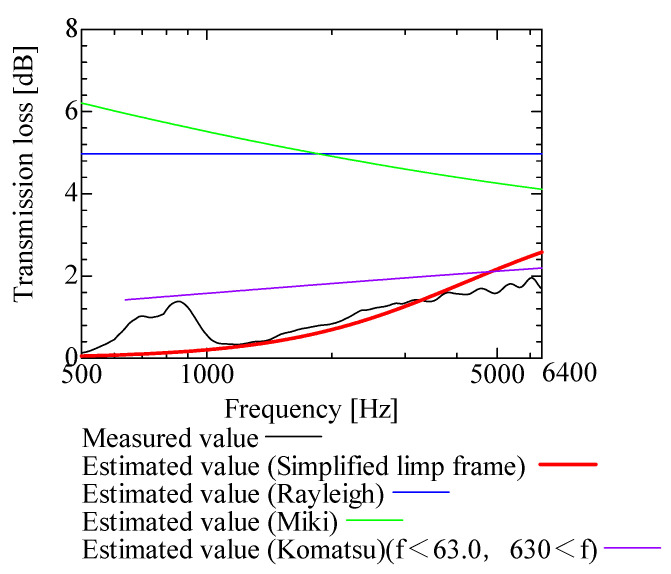
Comparison between experiment and estimates. Shinwa 2-3, m = 13.52 g/m^2^, t = 70 µm.

**Figure 30:**
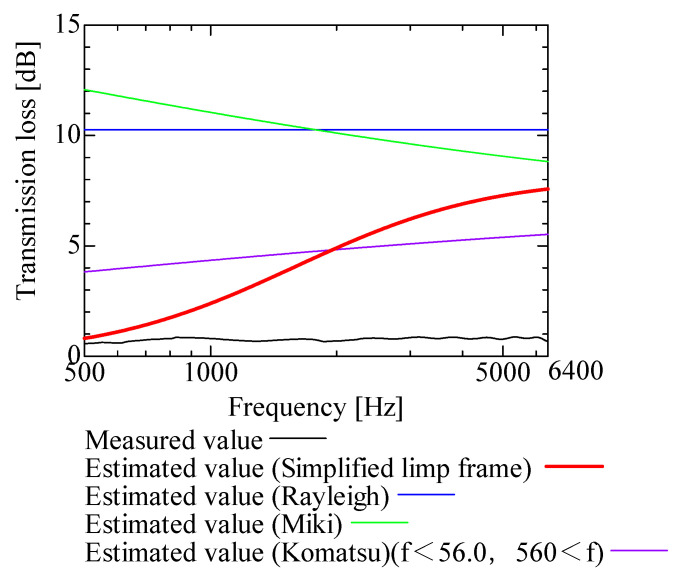
Comparison between experiment and estimates. Sample E, m = 80.3 g/m^2^, t = 230 µm. When the experimental values are <1 dB, none of the estimation models align with the experimental results.

**Figure 31:**
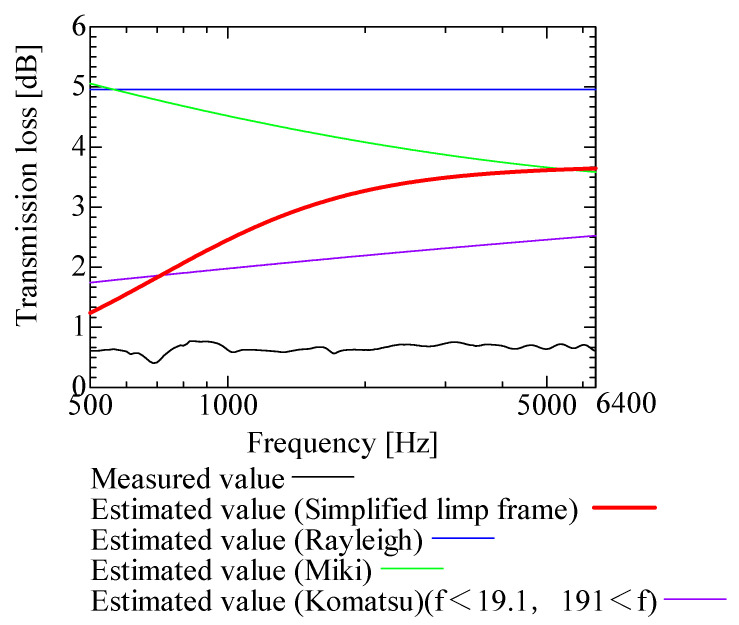
Comparison between experiment and estimates. Sample C, m = 80.06 g/m^2^, t = 230 µm. When the experimental values are <1 dB, none of the estimation models align with the experimental results.

**Figure 32:**
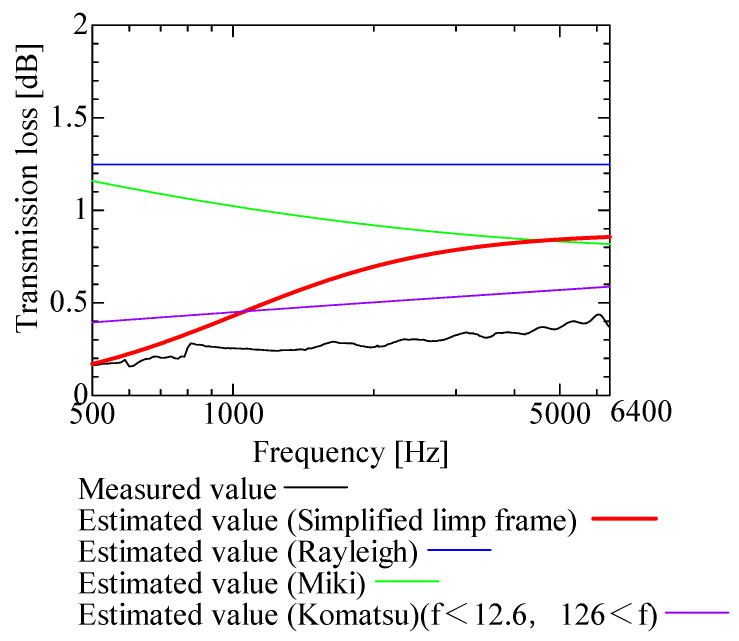
Comparison between experiment and estimates. Shinwa 2-4, m = 13.05 g/m^2^, t = 70 µm. When the experimental values are <1 dB, none of the estimation models align with the experimental results.

**Figure 33:**
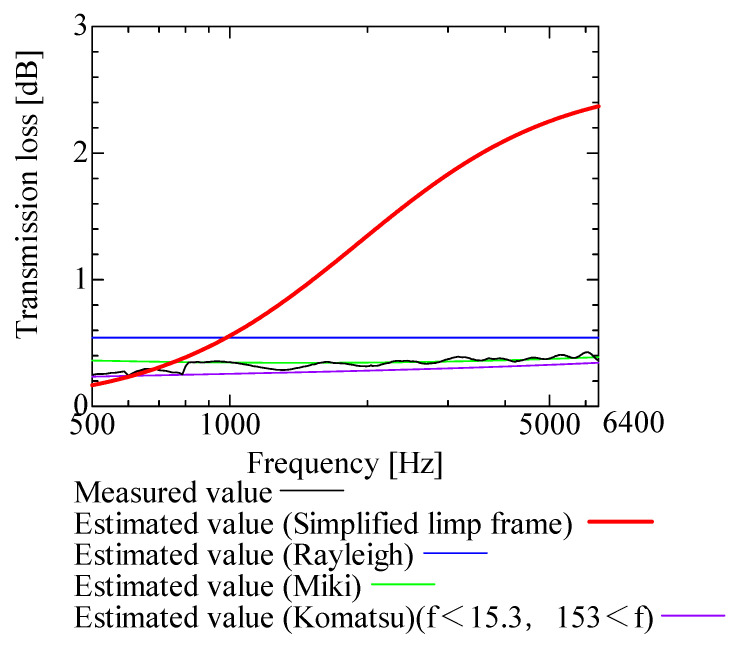
Comparison between experiment and estimates. Shinwa 1-4, m = 20.05 g/m^2^, t = 240 µm. The estimation models, except the SLFM, are consistent with the experimental data.

**Table 1:** Properties of test samples. Upper; Properties of test Samples (nanofibers). Lower; Properties of test Samples (base materials).

Properties of Test Samples (Nanofibers)					
	Thickness t [µm]	Area Density m [g/m^2^]	Flow Resistivity σ [Ns/m^4^]	Material (Nano/Substrate)	Corresponding Figure Number
Sample A	60	18.2	5.26 × 10^6^	PVDF/PET	26
Sample B	60	18.6	1.48 × 10^7^	PVDF/PET	23
Sample C	230	80.06	1.91 × 10^6^	PVDF/PET	31
Sample D	230	80.12	2.99 × 10^6^	PVDF/PET	29
Sample E	230	80.3	5.60 × 10^6^	PVDF/PET	30
Sample F	230	81.1	1.63 × 10^7^	PVDF/PET	14
Sample 1	56	18	2.55 × 10^7^	PESU/PET	19
Sample 2	2000	100	4.71 × 10^5^	PESU/PET	16
Shinwa 1-1	240	22	5.94 × 10^6^	PVDF/PP	20
Shinwa 1-2	240	21.2	2.98 × 10^6^	PVDF/PP	22
Shinwa 1-3	240	20.6	1.20 × 10^6^	PVDF/PP	27
Shinwa 1-4	240	20.05	1.53 × 10^6^	PVDF/PP	33
Shinwa 2-1	70	15.4	2.04 × 10^7^	PVDF/PP:PE	25
Shinwa 2-2	70	14.2	1.14 × 10^7^	PVDF/PP:PE	21
Shinwa 2-3	70	13.52	6.30 × 10^6^	PVDF/PP:PE	28
Shinwa 2-4	70	13.05	1.26 × 10^6^	PVDF/PP:PE	32
Tenma 1	99.4	34.1	9.74 × 10^6^	cellulose/PP	18
Tenma 2	122.5	49.3	1.16 × 10^7^	cellulose/PP	15
Tenma 3	104.3	25	7.68 × 10^7^	cellulose/PP	17
Tenma 4	85.7	20	4.27 × 10^7^	cellulose/PP	24
**Properties of Test Samples (Base Materials)**					
	**Thickness** t **[µm]**	**Area Density** m **[g/m^2^]**	**Flow Resistivity** **σ [Ns/m^4^]**		**Material (Nano/Substrate)**
Sample G	60	18	5.00 × 10^5^		PVDF/PET
Sample H	230	80	1.87 × 10^5^		PVDF/PET
Shinwa 1-5	240	20	7.93 × 10^4^		PVDF/PET
Shinwa 2-5	70	13	2.99 × 10^5^		PVDF/PET
Base of Sample 1	56	15	-		PVDF/PET

**Table 2:** Biot parameters for limp frame model (LFM).

	Biot Parameters	Variables	Value	Unit
Acoustical	Flow resistivity	σ	5.26 × 10^6^	Ns/m^4^
	Porosity	*ϕ*	0.78	
	Tortuosity	$\alpha_{\infty }$	1.1	
	Vicious characteristics length	Λ	4.8	µm
	Thermal characteristics length	$\Lambda^{\prime }$	5.5	µm
Structual	Bulk density	ρ	303	kg/m^3^

## Data Availability

Data are contained within the article.
